# ACTION trial: a prospective study on diagnostic **A**ccuracy of 4D **CT** for diagnosing **I**nstable Scaph**O**lunate Dissociatio**N**

**DOI:** 10.1186/s12891-021-03946-x

**Published:** 2021-01-15

**Authors:** Leonie Goelz, Simon Kim, Claas Güthoff, Frank Eichenauer, Andreas Eisenschenk, Sven Mutze, Ariane Asmus

**Affiliations:** 1 Dept. of Radiology and Neuroradiology, BG Unfallkrankenhaus, Berlin, Germany; 2 Inst. For Diagnostic Radiology, University Medicine Greifswald, Greifswald, Germany; 3 Dept. of Hand Surgery and Microsurgery, University Medicine Greifswald, Greifswald, Germany; 4 Center for Clinical Research, BG Unfallkrankenhaus, Berlin, Germany; 5 Dept. of Hand-, Replantation- and Microsurgery, BG Unfallkrankenhaus, Berlin, Germany

**Keywords:** Scapholunate ligament, Dynamic, 4D CT, Arthroscopy, Cineradiography, Diagnostic accuracy, Wrist

## Abstract

**Background:**

Early detection of scapholunate ligament (SLL) tears is essential after minor and major trauma to the wrist. The differentiation between stable and instable injuries determines therapeutic measures which aim to prevent osteoarthritis. Arthroscopy has since been the diagnostic gold standard in suspected SLL tears because non-invasive methods have failed to exclude instable injuries reliably. This prospective study aims to determine the diagnostic accuracy of dynamic, 4D computed tomography (CT) of the wrist for diagnosing instable SLL tears.

**Methods:**

Single center, prospective trial including 40 patients with suspected SLL tears scheduled for arthroscopy. Diagnostic accuracy of 4D CT will be tested against the reference standard arthroscopy. Radiologists will be blinded to the results of arthroscopy and hand surgeons to radiological reports. A historical cohort of 80 patients which was diagnosed using cineradiography before implementation of 4D CT at the study site will serve as a comparative group.

**Discussion:**

Static imaging lacks the ability to detect instable SLL tears after wrist trauma. Dynamic methods such as cineradiography and dynamic magnetic resonance imaging (MRI) are complex and require specific technical infrastructure in specialized centers. Modern super-fast dual source CT scanners are gaining popularity and are being installed gradually in hospitals and ambulances. These scanners enable dynamic imaging in a quick and simple manner. Establishment of dynamic 4D CT of the wrist in patients with suspected SLL tears in in- and outpatient settings could improve early detection rates. Reliable identification of instable injuries through 4D CT scans might reduce the number of unnecessary diagnostic arthroscopies in the future.

**Trial registration:**

This study was registered prospectively at the German Clinical Trials Register (DRKS) DRKS00021110. Universal Trial Number (WHO-UTN): U1111–1249-7884.

## Administrative information

Note: the numbers in curly brackets in this protocol refer to SPIRIT checklist item numbers. The order of the items has been modified to group similar items (see http://www.equator-network.org/reporting-guidelines/spirit-2013-statement-defining-standard-protocol-items-for-clinical-trials/).

**Table:** 

Title {1}	A Prospective Study on Diagnostic Accuracy of 4D CT for Diagnosing Instable ScaphOlunate DissociatioN (ACTION Trial)
Trial registration {2a and 2b}.	This study was registered prospectively at the German Clinical Trials Register (DRKS) DRKS00021110. Universal Trial Number (WHO-UTN): U1111–1249-7884. Registration date 05/12/2020. https://www.drks.de/drks_web/navigate.do?navigationId=trial. HTML&TRIAL_ID=DRKS00021110
Protocol version {3}	May 25th, 2020; Version 1.0
Funding {4}	The study site will be supported with 17.000€ by a nonprofit organization, the “Deutsche Arthrose-Hilfe” (German Osteoarthritis Support Group), during this trial.
Author details {5a}	**BG Klinikum Unfallkrankenhaus Berlin, Germany** Dr. Leonie Goelz - Dept. of Radiology and Neuroradiology Claas Güthoff - Center for Clinical Research Frank Eichenauer - Dept. of Hand-, Replantation- and Microsurgery Prof. Dr. Andreas Eisenschenk. - Dept. of Hand-, Replantation- and Microsurgery Prof. Dr. Sven Mutze - Dept. of Radiology and Neuroradiology Dr. Ariane Asmus - Dept. of Hand-, Replantation- and Microsurgery **University Medicine Greifswald, Germany** Dr. Leonie Goelz - Inst. for Diagnostic Radiology Dr. Simon Kim - Dept. of Hand Surgery and Microsurgery Prof. Dr. Andreas Eisenschenk - Dept. of Hand Surgery and Microsurgery Prof. Dr. Sven Mutze - Inst. for Diagnostic Radiology
Name and contact information for the trial sponsor {5b}	BG Klinikum Unfallkrankenhaus Berlin, Germany Depts. of Radiology and Neuroradiology/Hand-, Replantation- and Microsurgery Warener Straße 7, 12,683 Berlin, Germany Leonie.goelz@ukb.de
Role of sponsor {5c}	The study site will be supported with 17.000€ by a nonprofit organization, the “Deutsche Arthrose-Hilfe” (German Osteoarthritis Support Group), during this trial. Results will be published independent of any external influence.

## Introduction

### Background and rationale {6a}

Tears of the scapholunate ligament (SLL) are a common injury after wrist trauma [1]. Some partial ruptures of the ligament can be compensated, but undetected instable SLL tears usually lead to chronic wrist pain and disabling osteoarthritis [2]. The differentiation between stable and instable injuries influences therapeutic measures crucially. Stable SLL tears can be approached conservatively while complete or instable injuries should be repaired/stabilized [3]. Arthroscopy has long been the standard for any type of suspected SLL injury because results of diagnostic imaging are considered unreliable [4]. Thus, researchers have concentrated on developing methods to classify SLL tears noninvasively but dependably. In general, imaging of the SLL can be divided into static and dynamic techniques. Radiographs are the primary radiological method in cases of wrist pain but detect only static forms of SLL injuries accurately [5]. Through multi-slice CT and MRI of the carpal joints the SLL can be visualized directly. Direct arthrography improves accuracy of both methods from 56 to 100% [6, 7]. Early stages of SLL dissociation have been approached by dynamic methods such as cineradiography or cine MRI [5, 8]. Nevertheless, both arthrography and cineradiography are time-consuming; require specialized equipment, and personnel [9].

Evolution of CT technique towards dual source CT scanners as well as towards wider detectors paired with a higher number of detector rows offers another method for dynamic imaging paired with three-dimensional evaluations. Image quality of so called 4D CTs of the wrist has been described as excellent and inter-observer reliability for analysis of wrist kinematics seems promising [9–12]. Latest studies by Athlani et al. and de Roo et al. [13, 14] have investigated the SL gap with 4D CT in healthy and injured individuals. de Roo et al. presented a custom made device to standardize evaluation of the SL joint during radioulnar deviation [15]. Even though this approach improves the reproducibility of results, a custom made device will hardly be transferable to everyday routine. Both studies are lacking a diagnostic gold standard to compare the results of 4D CT. Nevertheless, 4D CT is described as feasible and its results helpful in differentiating SL instability.

Currently prospective data discussing the diagnostic accuracy of 4D CT of the wrist for classifying SLL tears is not available. Establishment of dynamic 4D CT of the wrist in patients with suspected SLL tears in in- and outpatient settings could improve early detection rates of this injury. Reliable identification of instable injuries through 4D CT scans might reduce the number of unnecessary diagnostic arthroscopies in the future.

### Objectives {7}

Primary objectives:

1. Analysis of diagnostic accuracy of 4D CT for the differentiation between stable and instable SLL tears preoperatively.
2. Comparison of diagnostic accuracies of 4D CT and cineradiography.

*Secondary objectives:*

- Can 4D CT scans of the wrist detect secondary injuries as opposed to radiography?
- How do the stages of scapholunate dissociation correlate with the time interval between diagnostics and trauma?
- Are measured effective radiation skin doses in-vivo and calculated skin doses during 4D CT similar?
- Which diagnostic algorithm can be proposed for suspected SLL tears?

### Trial design {8}

Prospective, exploratory study of diagnostic accuracy of 4D CT with arthroscopy as reference test adhering to the Standards for Reporting of Diagnostic Accuracy Studies (STARD) guidelines [15].

Additional comparison of sensitivity and specificity of 4D CT in detecting instable SLL tears to that of cineradiography observed in a historical cohort.

## Methods: participants, interventions and outcomes

### Study setting {9}

Single center study at an urban trauma hospital with a dedicated Department of Radiology specialized in musculoskeletal injuries and a Department of Hand-, Replantation- and Microsurgery located in Berlin, Germany.

### Eligibility criteria {10}

Eligible patients for prospective inclusion with suspected SLL tear are scheduled for diagnostic arthroscopy. 40 consecutive patients will be enrolled according to predefined criteria:

- Inclusion criteria: patients > 18 years, written consent.
- Exclusion criteria: inability to consent, patients < 18 years, pregnancy, considerably limited range of radio-ulnar motion, incompliance to the rehearsed radio-ulnar motion, scapholunate advanced collapse (SLAC), previous carpal instability or carpal operation, incomplete historical records.
  - Patients with distal radius fractures and previous stabilization of the fracture will not be excluded per se. Only if these patients are not able to perform the required radio-ulnar motion due to pain or stiffness, they will be excluded. Patients with previous carpal instabilities or carpal operations i.e. due to scaphoid fractures will be excluded from this study as well.

A historical cohort consisting of 80 consecutive patients with the same inclusion and exclusion criteria who were diagnosed with cineradiography preoperatively during the pre-4D CT era will serve as comparison for the diagnostic value of the new method.

Two radiologists with more than 6 years of experience in musculoskeletal radiology will evaluate preoperative 4D CT scans and cineradiographies independently.

Arthroscopies will be performed by specialized hand and wrist surgeons.

### Who will take informed consent? {26a}

Written consent will be obtained by the attending hand surgeon or radiologist.

### Additional consent provisions for collection and use of participant data and biological specimens {26b}

Patients eligible for retrospective inclusion will be notified and informed about the study and asked for written consent via mail by the study center.

### Interventions

#### Explanation for the choice of comparators {6b}

The newly established imaging technique 4D CT has to be evaluated and tested for diagnostic accuracy. The diagnostic gold standard arthroscopy will serve as the reference test and prevent therapeutic gaps in case of inaccurate preoperative diagnosis.

For comparative results an additional evaluation of preoperative cineradiograms, the previous diagnostic/imaging standard of the study center, will be enclosed in a retrospective approach. Classifications of SLL injuries on cineradiograms will also be evaluated against the results of arthroscopy.

#### Intervention description {11a}

All patients with a typical trauma mechanism and wrist pain presenting through the ambulance or emergency room will be screened for eligibility. Patients will be examined and will receive radiographs of the wrist in two planes routinely. In cases of suspected SLL tear and indication for arthroscopy, patients will be informed about the possibility of study participation by the attending hand surgeon.

According to the inclusion and exclusion criteria 40 participants will be enrolled consecutively after written consent.

4D CT scans of the injured wrist will be performed preoperatively and analyzed by two specialized radiologists. Coronal reconstructions will be used to measure the maximum and minimum scapholunate distance (SLD). On sagittal reconstructions the scapholunate (SL) and radiolunate (RL) angle will be determined to classify SLL tears as stable or unstable.

The patients will not be exposed to radiation for research purposes. Arthroscopy will be performed as the diagnostic gold standard within the same week to classify the type of SLL tear according to the European Wrist Arthroscopy Society (EWAS) and Geissler [16, 17]. Primary reconstruction and temporary arthrodesis will be performed when necessary, but the surgical therapy will not be part of the study.

We will conduct an additional screening of historical patient records for eligibility, inclusion, and exclusion criteria. These historical patients with a typical trauma and wrist pain were also examined clinically and received radiographs of the wrist in two planes routinely. In cases of suspected SLL tear, cineradiography was performed before arthroscopy. After written consent 80 consecutive patient who were diagnosed through cineradiography before arthroscopy will be enrolled. Measurement of minimum and maximum SLD on anterior-posterior projections, of SL angle, and RL angle on lateral projections will be measured to classify SLL tears as stable or unstable and to compare these results to the findings of arthroscopy. The diagnostic accuracy of cineradiography will serve as a comparison for the sensitivity and specificity of 4D CT in suspected SLL tears.

#### Criteria for discontinuing or modifying allocated interventions {11b}

Eligible patients who are incapable of performing the radio-ulnar motion required for dynamic evaluation of the SLL will be excluded from the study. Patients who withdraw their consent for arthroscopy will be excluded from the study.

Patients will be able to withdraw their consent for participation at any time during the trial. Patients with incomplete historical records will be excluded from retrospective analysis.

#### Strategies to improve adherence to interventions {11c}

Participants will be informed about the indication for arthroscopy irrespective of study participation. Close examination and clinical testing will reduce the number of patients who will have to be excluded due to an inability to perform the required radio-ulnar motion.

#### Relevant concomitant care permitted or prohibited during the trial {11d}

Not applicable to this diagnostic study.

#### Provisions for post-trial care {30}

As a purely observational study, participants will receive standard imaging/diagnostics and care during the trial. No study specific radiation exposure, clinical examinations, therapies, or follow-up exams will be conducted. Patients will be insured through the hospital’s liability insurance.

#### Outcomes {12}

Measurements on 4D CT reconstructions and cineradiography 1–7 days preoperatively will be used as index tests [Table 1] to calculate diagnostic accuracies of both methods for detecting instable SLL tears as stipulated by the reference test arthroscopy.

**Table 1:** Radiological classification of stability

Stability	SLD_**min**_ (mm)	SLD_**max**_ (mm)	SL °	RL °
stable	< 3	< 3	< 60	> −15
instable	< 3 or > 3	> 3	> 60	> −15 or < − 15

SL distance = *SLD*, radiolunate= *RL*, scapholunate SL angle)

Binary classification of therapeutic indication to conservative therapy vs surgery determined by arthroscopy will be used as reference test to classify SLL injuries [Table 2].

**Table 2:** Arthroscopic classification of stability

Stability	Stadium (Geissler/ EWAS)	Anatomical Correlation	Arthroscopic test
stable	I	elongation	Microhook cannot be advanced into the joint
	II	rupture of membranous portion of SLL	Microhook can be advanced into the joint but joint is not widened
unstable	IIIA	II + rupture of palmar portion of SLL	Joint widens on the palmar side during manipulation with microhook
	IIIB	II + rupture of dorsal portion of SLL	Joint widens on the dorsal side during manipulation with microhook
	IIIC	II + rupture of palmar and dorsal portion	Joint widens on both palmar side during manipulation with microhook but closes after manipulation
	IV	IIIC + widening of SL joint	Joint can be passed by arthroscope
	V	IV + tilting of carpalia	widening of SL joint with pathological plain radiographs

Validity will be described by sensitivity and specificity of a diagnostic test [18]. Both index tests, 4D CT and cineradiography, will be opposed to the reference test arthroscopy. Optimal cut-off points are determined using area under the receiver operating characteristic (AUC-ROC) curves [19].

Effective skin dose measurements will be enabled using thermoluminescent dosimeters (TLD) during 4D CT at defined regions of interest (head, affected hand and shoulder).

#### Participant timeline {13}

**Fig. 1:**
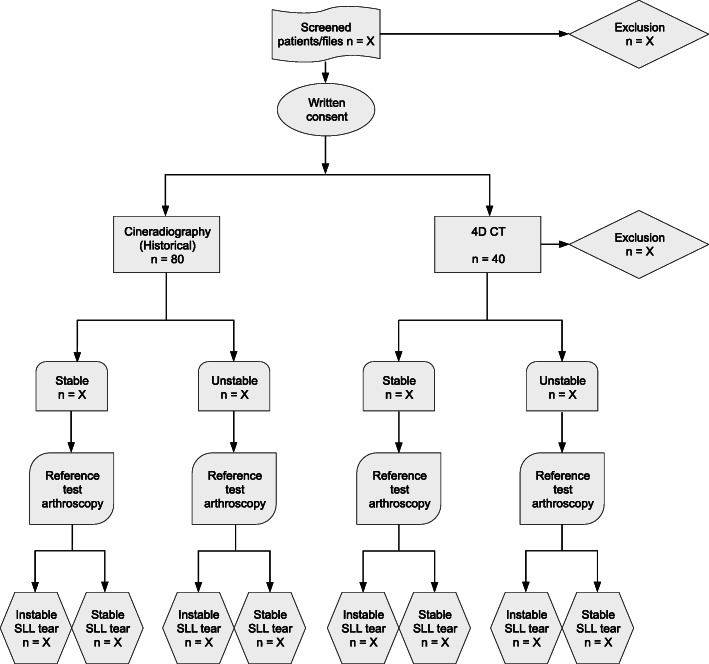
Flow diagram to test diagnostic accuracy of 4D CT and cineradiography in comparison

Table 3 describes the stages of the whole study period. Figure 1 illustrates the participant flow from screening to reference testing.

**Table 3:** STUDY PERIOD

	Enrolment	***Allocation***	Observational period			Close-out
**TIMEPOINT**	-t_1_	Not applicable	t_1_ (CT-imaging/inspection of cineradiography reports)	t_2_ (arthroscopy/inspection of surgical reports)	t_3_ (discharge)	t_x_ (statistical analysis index test vs. reference test, see flow chart)
**ENROLMENT:**						
Eligibility screening (patients/files)	X					
Informed consent	X					
**STUDY GROUPS:**						
4D CT (prospective)			x	x	x	x
Cineradiography (retrospective)			x	x	x	x
**ASSESSMENTS:**						
Stability			x	x		
Effective skin dose 4D CT			x

#### Sample size {14}

The study will be conducted and analyzed in an exploratory fashion. Therefore, formal sample size calculations are deferred by the investigators. Instead, yearly patient numbers were estimated, and the number of participants was adapted to a two-year study design which results in a recruiting target of 40 patients used for estimation of diagnostic accuracy of 4D CT. The comparative historical cohort used for estimation of diagnostic accuracy of cineradiography should consist of 80 patients to account for a probably greater extent of heterogeneity in the retrospective data due to the fact that these were collected by different clinicians who were not instructed on data collection specifically for this trial.

#### Recruitment {15}

Previously, approximately 30 arthroscopies have been performed at the study center for suspected SLL tears each year. Thus, enrolment of 40 patients in 2 years seems reasonable. To assure maximum screening and identification of eligible patients, all hand surgeons at the center will be involved in the process. Radiologists are sensitized to communicate radiographs with abnormalities of the SL joint to increase the screening cohort.

### Assignment of interventions: allocation

#### Sequence generation {16a}

The prospective part of the trial is a single arm observational study of diagnostic accuracy of 4D CT.

A historical cohort serves as a comparative group diagnosed through cineradiography preoperatively. The switch of the diagnostic/imaging standard at the study center from cineradiography to 4D CT for diagnosing instable SLL tears results in allocation to the historical and prospective group. Randomization is not part of this trial.

#### Concealment mechanism {16b}

Not applicable.

#### Implementation {16c}

Patients will be enrolled prospectively by attending hand surgeons and radiologists.

### Assignment of interventions: blinding

#### Who will be blinded {17a}

Hand surgeons will be blinded to the results of 4D CT and radiologists will be blinded to the results of arthroscopies. Both departments will supply the statistician with their results.

Radiologists were unaware of the results of arthroscopy while reporting cineradiograms prior to surgery but hand surgeons were aware about imaging results preoperatively.

#### Procedure for unblinding if needed {17b}

Unblinding of attending physicians is not permissible. Patients will receive the therapeutic standard which is guaranteed by arthroscopy independent of the results of 4D CT.

### Data collection and management

#### Plans for assessment and collection of outcomes {18a}

Hand surgeons will be trained to examine patients during screening in a standardized fashion. Arthroscopies will be performed with the patient under general or regional anesthesia. At first, the membranous or proximal part of the SLL complex is visualized and the tear located through a 3/4 and 5 U portal. The SLL will be examined using a 2.4-mm arthroscope with a 30° wide angle lens (Storz, Erlangen, Germany) and probed with a hook. A tear will be differentiated into an avulsion from the lunate or scaphoid or a midsubstance ligament rupture. Secondly, through the midcarpal ulnar (MCU) and midcarpal radial (MCR) portals the SLL tear will be classified. Surgical reports will be descriptive, but they will include a final EWAS/Geissler classification to describe stability of the SL joint on a case report form (CRF) [16, 17]. The following data will be documented by hand surgeons on paper CRFs [Table 4].

**Table 4:** Case Report Form

Patient-ID:		
**Screening:** Date		
Suspected SLL tear	Yes	No
Indication for arthroscopy	Yes	No
> 18 Years	Yes	No
Ability to perform radio-ulnar motion	Yes	No
Written informed consent	Yes	No
**Exclusion criteria:** Date		
Pregnancy	Yes	No
Incompliance for radio-ulnar motion		
SLAC	Yes	No
**Enrolment:** Date		
Date of enrolment		
**Epidemiology:**		
Gender	Male	Female
Side of injury	Right	Left
Handedness	Right	Left
Date of injury		
Age at the day of injury		
**Clinical examination:** Date		
Watson-Test	Positive	Negative
Localized pain during compression of dorsal SLL	Yes	No
**Arthroscopy:** Date		
Age at the day of arthroscopy		
EWAS/Geissler Stadium		
Stability	Stable	Unstable

4D CTs will be acquired using a dual-source scanner with 2 × 192 rows and a detector width of 6 cm (Somatom Force, Siemens, Germany). Patients will be placed in prone position with the injured wrist reaching into the gantry (“superman position”). Patients will be instructed to perform a radio-ulnar movement during an 8 s scan cycle. Forearms will only be lightly fixated with a tape as a reminder for the patients to reside to wrist movements only. Postprocessing will be performed using a designated software (Syngo.Via Version VB10B-VB30A, Siemens, Germany). Reconstructions include dynamic 5 mm and 1 mm coronal reconstructions as well as static coronal and sagittal reconstructions of 2 mm. Table 5 summarizes the acquisition parameters of 4D CT.

**Table 5:** Parameters of 4D CT

Tube voltage (kV)	80
Milliamperage (mAs)	70
Section thickness (mm)	1
Length (mm)	56
Number of slices	1254
Collimation	384 × 1 mm
Reconstruction matrix	Soft-tissue and bone kernel
Image reconstruction	512 × 512

Radiologists will document results of 4D CT in a standardized manner to describe stability of the SLL ([20], Table 6). SLD will be measured in the center of the SL joint during radio-ulnar motion at minimum and maximum width [21, 22]. Carpal angles will be measured on sagittal reconstructions according to the radial axis [23, 24].

**Table 6:** Radiological Report

Patient-ID:		
**Parameter:** Date		
Modality	4D CT	Cineradiography
Date of imaging		
SLD_min_		
SLD_max_		
SL angle		
RL angle		
**Stability**	Stable	Unstable
**Effective skin dose of 4D CT**		
1 ipsilateral hand		
2 ipsilateral shoulder		
3 head		
CT dose index		
Dose length product		

### Plans to promote participant retention and complete follow-up {18b}

This observational study will report on diagnostic accuracies of 4D CT and cineradiography. The observation will end with discharge. Follow-up examinations will not be part of this study. Plans to promote participant retention are not required.

### Data management {19}

Data will be collected on paper CRFs and stored in the study center in each department for the duration of the study and 5 years thereafter. Paper CRFs will be copied into digital files in excel format and protected by departmental passwords. Data will be checked for completeness during digitalization. Only one of the attending physicians of each department is selected for data management and digitalization. Digitally secured data will be transferred on hard drives to the statistician at the end of data collection.

### Confidentialitesy {27}

Patient information will be collected and processes only by attending physicians. For study purposes, patient information will be pseudonymized during analysis by each department independently. The lists for decoding pseudonyms will be kept in a password secured and firewall protected PC at each department. The statistician will possess a master key to match pseudonyms of each department to recombine the participants results for analysis. Radiologists and hand surgeons will collect data separately on paper CRFs. Data will be entered into digital tables secured with departmental passwords and checked for completeness. The statistician will receive data from both departments separately for analysis. Study data won’t leave the study center. Publication of anonymous data independently of the study results is planned.

### Plans for collection, laboratory evaluation and storage of biological specimens for genetic or molecular analysis in this trial/future use {33}

Not applicable.

### Statistical methods

#### Statistical methods for primary and secondary outcomes {20a}

All statistical analyses will be conducted in an exploratory, hypotheses-generating fashion. For both index tests AUCs as well as sensitivity and specificity with 95% confidence intervals will be calculated. Descriptive statistics dependent on scale and distribution will be reported for all parameters.

#### Interim analyses {21b}

Not applicable.

#### Methods for additional analyses (e.g. subgroup analyses) {20b}

Interobserver agreement for cineradiographies and 4D CT between two radiologists will be evaluated using the kappa statistic.

#### Methods in analysis to handle protocol non-adherence and any statistical methods to handle missing data {20c}

One exclusion criterium for the historical cohort will be missing data. The comparative historical cohort will consist of 80 patients to account for a probably greater extent of heterogeneity in the retrospective data.

#### Plans to give access to the full protocol, participant level-data and statistical code {31c}

The full protocol, anonymized participant level-data, and statistical code can be provided upon request.

### Oversight and monitoring

#### Composition of the coordinating center and trial steering committee {5d}

This single center, investigator-initiated study will not be monitored independently. Each department will be responsible for the integrity of the data. One physician of each department is selected for data management and digitalization. After discharge of all 40 participants and protocolling of the data of all 80 patient files the responsible hand surgeon and radiologist will transfer pseudonymized separate data sets to the statistician. The statistician will receive both data sets and will be the only one with access to the final data set.

#### Composition of the data monitoring committee, its role and reporting structure {21a}

The data monitoring committee will consist of one radiologist, one hand surgeon, and the statistician of the study center. After data acquisition will be complete, integrity of digital data will be cross-checked by hand surgeons and radiologist using the paper CRFs. The statistician will be the only member with insight to the finalized combined dataset. There will be no external monitor.

#### Adverse event reporting and harms {22}

The threshold for acute stochastic skin damage has been reported to lie around 2 Gy [25] and thus almost 60x higher than the skin doses of reports about 4D CT of the wrist in-vivo and in-vitro [12, 26]. Nevertheless, concerns about deterministic cells damages by low-dose examinations arise recurrently. Radiation exposure is measured during 4D CT and checked for compliance with local regulations (https://www.bfs.de). Violations have to be reported (https://www.aerztekammer-berlin.de/10arzt/39_Aerztliche_Stelle/05_AESQSB_kurz_dargestellt/index.html).

#### Frequency and plans for auditing trial conduct {23}

Audits will not be part of this trial.

#### Plans for communicating important protocol amendments to relevant parties (e.g. trial participants, ethical committees) {25}

Amendments to the study protocol will be reported to the ethics board at the University Medicine Greifswald, Germany (http://www2.medizin.uni-greifswald.de/ethik/). Amendments cannot affect the standard of care; therefore, participants won’t be informed about changes. The investigators will meet once every 3 months to discuss protocol relevant issues.

#### Dissemination plans {31a}

This study will be published open-access in a peer-reviewed journal independent of the results. Prior to publication, results will be available on a preprint server for transparency of any modifications of the manuscript.

## Discussion

Studies of diagnostic accuracy have the potential to improve the standard of medical care. Well conducted studies which are reported adhering to standardized guidelines allow for a generalizability of the topic [15]. Observational studies have the advantage of answering certain scientific questions in a rather descriptive manner while excluding any harm induced by study specific interventions. The lack of direct comparisons through a strict randomization protocol is a disadvantage of this study type.

On these grounds it can be expected that this prospective observational study will result in reliable calculations of diagnostic parameters of 4D CT of the wrist in diagnosing instable SLL tears. The comparison with a historical cohort on the other hand, will always be a means to atone for missing randomization and prospective comparisons.

In order not to over-estimate the clinical relevance of 4D CT, it will be crucial to minimize drop-outs after conducting the index test. The study protocol requires that all patients will have to have an indication for the reference test arthroscopy first to be eligible as participants. The number and reasons for drop-outs and excluded patients will be described in detail in the study report.

It can be expected that a positive outcome of the study and an increasing availability of 4D CT could have a grave impact on out-patient care. Since approximately 5% of all wrist sprains have an associated injury of the SLL [27], a broad availability of reliable primary imaging might improve early detection rates, reduce the incidence of resulting osteoarthritis, and the number of unnecessary diagnostic arthroscopies in the future.

### Trial status

Protocol Version 1.0. Recruitment of first participant: 06/16/2020. Recruitment will be completed 06/2022.

## Data Availability

The final anonymized trial dataset can be supplied upon personal request. Detailed statistical analysis will be included in the published article as supplementary information files.

## Abbreviations

CT: Computed tomography
MRI: Magnetic resonance imaging
SLL: Scapholunate ligament
SLAC: Scapholunate advanced collapse
SLD: Scapholunate distance
SL: Scapholunate
RL: Radiolunate
CRF: Case report form
EWAS: European Wrist Arthroscopy Society
TLD: Thermoluminescence dosimeter
AUCs: Areas under the curve
DLP: Dose length product
CTDI: CT dose index
AUROC: Area under the receiver operating characteristic
STARD: Standards for Reporting of Diagnostic Accuracy Studies
MCU: Midcarpal ulnar
MCR: Midcarpal radial
